# P Wave Area for Quantitative Electrocardiographic Assessment of Left Atrial Remodeling

**DOI:** 10.1371/journal.pone.0099178

**Published:** 2014-06-05

**Authors:** Jonathan W. Weinsaft, Jonathan D. Kochav, Jiwon Kim, Sergey Gurevich, Samuel C. Volo, Anika Afroz, Maya Petashnick, Agnes Kim, Richard B. Devereux, Peter M. Okin

**Affiliations:** 1 Greenberg Cardiology Division/Department of Medicine, Weill Cornell Medical College, New York, New York, United States of America; 2 Department of Radiology, Weill Cornell Medical College, New York, New York, United States of America; 3 Memorial Sloan Kettering Cancer Center Department of Medicine, New York, New York, United States of America; 4 Duke University School of Medicine, Durham, North Carolina, United States of America; University of Perugia, Italy

## Abstract

**Background:**

Left atrial (LA) dilation provides a substrate for mitral regurgitation (MR) and atrial arrhythmias. ECG can screen for LA dilation but standard approaches do not assess LA geometry as a continuum, as does non-invasive imaging. This study tested ECG-quantified P wave area as an index of LA geometry.

**Methods and Results:**

342 patients with CAD underwent ECG and CMR within 7 (0.1±1.4) days. LA area on CMR correlated best with P wave area in ECG lead V1 (r = 0.42, p<0.001), with lesser correlations for P wave amplitude and duration. P wave area increased stepwise in relation to CMR-evidenced MR severity (p<0.001), with similar results for MR on echocardiography (performed in 86% of patients). Pulmonary arterial (PA) pressure on echo was increased by 50% among patients in the highest (45±14 mmHg) vs. the lowest (31±9 mmHg) P wave area quartile of the population. In multivariate regression, CMR and echo-specific models demonstrated P wave area to be independently associated with LA size after controlling for MR, as well as echo-evidenced PA pressure. Clinical follow-up (mean 2.4±1.9 years) demonstrated ECG and CMR to yield similar results for stratification of arrhythmic risk, with a 2.6-fold increase in risk for atrial fibrillation/flutter among patients in the top P wave area quartile of the population (CI 1.1–5.9, p = 0.02), and a 3.2-fold increase among patients in the top LA area quartile (CI 1.4–7.0, p = 0.005).

**Conclusions:**

ECG-quantified P wave area provides an index of LA remodeling that parallels CMR-evidenced LA chamber geometry, and provides similar predictive value for stratification of atrial arrhythmic risk.

## Introduction

Left atrial (LA) chamber remodeling provides a physiologic substrate for mechanical and electrical alterations in cardiac performance. In population-based studies, LA size has been linked to clinical event risk, including heart failure and arrhythmias. [1]–[2] LA size has been shown to stratify prognosis for incident heart failure and mortality, with event risk independent of clinical indices. [3]–[4] Atrial fibrillation has also been closely linked to LA size,[5]–[7] consistent with the concept that LA remodeling alters electrical conduction and provides a physiologic substrate for arrhythmic events. Taken together, these findings provide a rationale for assessment of LA remodeling in at-risk patient populations.

Electrocardiography (ECG) is widely utilized as an initial screening test for patients with known or suspected heart disease. Unlike non-invasive imaging modalities (which directly measure LA geometry), ECG assesses LA physiology in relation to altered atrial electrical conductivity known to occur with chamber remodeling. P wave morphology is an established marker of atrial remodeling that has been used as an index of LA size for over a century. [8] However, conventional P wave criteria employ binary cutoffs for presence or absence of LA dilation,[9]–[11] rather than quantitative approaches that measure LA size as a continuum. Recent advances in ECG technology have facilitated quantification of constitutive components of the ECG waveform. Data from our group and others have shown quantitative analysis of the QRS complex to yield incremental utility over standard ECG approaches for assessment of LV remodeling and myocardial infarction. [12]–[13] P wave size and morphology can also be quantified in a similar manner, providing a quantitative index of LA remodeling that can be readily measured using standard data from the routine surface ECG.

This study tested the utility of P wave quantification as an index of LA remodeling among a broad cohort of subjects with ischemic heart disease. There were three aims: (1) to determine ECG based manifestations of LA chamber dilation as measured on cardiac magnetic resonance (CMR) imaging; (2) to identify alterations in P wave area that occur in relation to increasing magnitude of mitral regurgitation (MR) and pulmonary arterial hypertension; and (3) to test the predictive value of P wave area for stratification of longitudinal clinical endpoints related to LA remodeling – atrial fibrillation (AF) and atrial flutter (AFl).

## Methods

### Ethics Statement

The study protocol was approved by the Weill Cornell Institutional Review Board. For all subjects undergoing CMR and ECG exclusively for research purposes, written informed consent for study participation was obtained prior to data acquisition. For subjects meeting eligibility criteria but undergoing CMR and ECG for exclusively clinical purposes, data was retrospectively anonymized, with written informed consent for use of anonymized data waived.

### Population

The study population comprised subjects with coronary artery disease (CAD) undergoing ECG and CMR within a 1 week interval at Weill Cornell Medical College. CMR was performed in both post-MI and heart failure (LVEF≤40%) subjects undergoing CMR for assessment of LV function and remodeling. In all subjects, CAD was defined as ≥70% stenosis of a major epicardial vessel (or ≥50% stenosis of the left main) based on invasive angiography. Patients with incomplete cine-CMR exams (i.e., with respect to LA imaging) were excluded from study participation.

ECG and CMR were performed at Weill Cornell Medical College (New York, NY) from 2005 to 2013. Transthoracic echocardiography (echo), if acquired within 1 week of ECG, was used as a supplemental test to measure MR severity and pulmonary arterial pressures, for which results were obtained via query of institutional databases. Baseline demographic indices were obtained using a standardized patient questionnaire, with results supplemented by review of electronic medical records (EMR). Clinical follow-up (concerning incident AF or AFl) was obtained from EMR record review, which was performed by a dedicated study investigator blinded to results of both ECG and imaging. Adequate follow-up was defined based on documented clinical evaluation occurring at least 60 days after ECG.

### Data Acquisition

#### Electrocardiography

ECGs were acquired using a standard 12-lead surface electrode recording system (MAC 5000/5500, General Electric, Waukesha, WI). Recordings were performed at a sampling rate of 500 Hz. Data was stored digitally using a dedicated (GE Muse) archival system.

#### Cardiac magnetic resonance

CMR was performed by dedicated technologists using 1.5 Tesla scanners (General Electric, Waukesha, WI). Imaging consisted of two components: (1) cine-CMR for geometry/function, and (2) DE-CMR for myocardial infarction. Cine-CMR was performed using a steady-state free precession sequence (typical parameters: repetition time (TR) 3.5 msec, echo time (TE) 1.6 msec, flip angle 60°, in-plane spatial resolution 1.9 mm×1.4 mm). Following cine-CMR, gadolinium was intravenously administered (0.1–0.2 mmol/kg) to patients without renal contraindications. Delayed enhancement (DE-) CMR was performed 10–30 minutes thereafter using an inversion recovery pulse sequence (typical TI 250–350 msec).

Cine- and DE-CMR images were obtained in matching short and long-axis planes. Short-axis images were acquired throughout the LV from the mitral valve annulus through the apex. Long-axis images were acquired in standard two-, three- and four-chamber orientations.

#### Echocardiography

Transthoracic 2D echocardiograms were performed by experienced sonographers using commercially available equipment (e.g., General Electric Vivid-7, Philips ie33). Imaging was performed in parasternal, subcostal, as well as apical 2-, 3-, 4-, and 5- chamber orientations. Color Doppler was used to assess MR severity based on jet area, depth, vena contracta, and directionality. Pulsed wave Doppler included assessment of MR duration as well as pulmonary vein flow profiles.

### Data Analysis

#### P wave assessment

**Figure 1:**
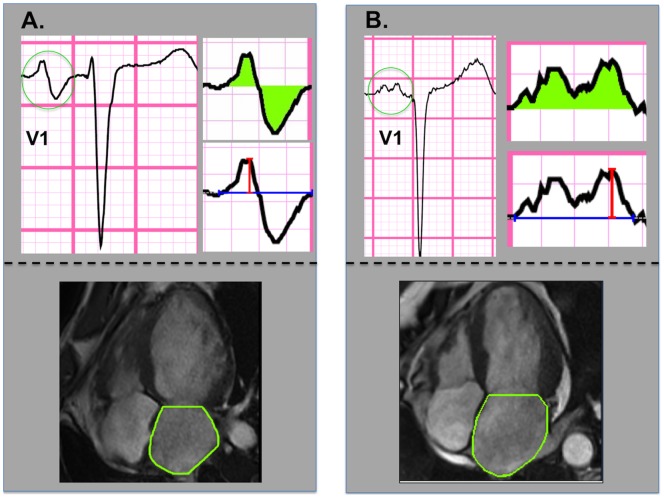
P wave and Left Atrial Area Quantification. Representative examples of LA quantification methods: For ECG (top), total P wave area was quantified based on geometric area (green shading) between the electrical waveform and the isoelectric line. Corresponding indices of amplitude (red line) and duration (blue line) were measured within the total P wave complex, as well as its positive and negative components. For CMR (bottom), LA area was measured by planimetry of chamber borders (green line) at ventricular end-systole. Note heterogeneity in P wave morphology among patients with LA enlargement: Whereas both patient examples demonstrate LA dilation by CMR, a bimodal P wave with large negative terminal component is present in **1A**, whereas a bifid but upright P wave is present in **1B**.

ECGs were quantitatively analyzed using automated processing software (Magellan, GE Healthcare, Wauwatosa, WI, USA). Multiple aspects of P wave morphology in lead V1 were measured, including duration, amplitude, total geometric area subtended within the waveform (inclusive of both positive and negative deflections) and the area of the terminal negative component of the P wave (**Figure 1** **)**.

#### Cardiac structure and function

CMR and echo indices of cardiac structure and function were measured to determine relationships between P wave characteristics and cardiac chamber remodeling. For cine-CMR, LA area was planimetered in 4-chamber orientation at ventricular end-systole (atrial end-diastole); left ventricular ejection fraction (LVEF), myocardial mass, and chamber volumes were measured via planimetry of contiguous short-axis images at ventricular end-diastole and end-systole. Corresponding echo measurements of LA and LV size were based on linear chamber dimensions in parasternal long axis views in accordance with consensus guidelines [14].

#### Mitral regurgitation

MR severity was graded on cine-CMR and echo in accordance with established conventions:

On cine-CMR, MR was graded based on maximal size of the regurgitant jet in relation to the left atrium (<1/3, 1/3–2/3, >2/3), with regurgitant severity scored using a 4 point scale (0 = none/trace, 1 = mild, 2 = moderate, 3 = severe) [15]–[16].

Echo was used as an additional test for MR, which was graded using a semi-quantitative 5-point scale. In accordance with established standards, [17] MR was primarily graded based on the farthest distance reached from the mitral orifice by the regurgitant jet (mild [1+]; ≤1.5 cm | moderate [2+]; 1.5–3.0 cm | moderately-severe [3+]; 3.0–4.5 cm | severe [4+]; ≥4.5 cm). MR severity was further confirmed based on jet area and vena contracta ([1+]; color jet <20% LA area, vena contracta <0.3 | [2+]; color jet 20–40%, vena contracta 0.3–0.69 | [3–4+]; color jet >40%, vena contracta ≥0.7) as well as jet density, mitral (E/A) and pulmonary vein flow pattern. [18] Pulmonary artery systolic pressure was calculated based on tricuspid valve regurgitant velocity and inferior vena cava caliber.

All imaging exams (both CMR and echo) were interpreted by experienced (ACC/AHA level III) investigators blinded to the results of ECG analysis.

### Statistical Methods

Continuous variables (expressed as mean±standard deviation) were compared using paired Student’s t-test for two-group comparisons. For multiple group comparisons, analysis of variance (ANOVA) was employed, as well as post-hoc (Tukey’s) testing for comparison of differences between individual strata. Categorical variables were compared using Chi-square or, when fewer than 5 expected outcomes per cell, Fisher’s exact test. ECG and imaging parameters were compared using bivariate correlation coefficients, with correlations further evaluated using Koenker’s test for heteroscedasticity. Multivariate logistic regression was used to test clinical and imaging indices in relation to ECG parameters. Follow-up event rates were compared between groups using Kaplan-Meier survival curves and Cox regression analysis. Two-sided p<0.05 was considered indicative of statistical significance. Statistical calculations were performed using IBM SPSS Statistics Version 20 (IBM Corporation, Armonk, NY).

## Results

### Population Characteristics

The study population comprised 342 subjects with ischemic heart disease who underwent CMR and ECG within a 1-week interval (mean 0.1±1.4 days); 94% had CMR and ECG performed within 1 day.

**Table 1** details clinical and imaging characteristics of the population, stratified in relation to presence or absence of CMR-evidenced LA dilation (categorized based on an established CMR cutoff [15 cm^2^/m^2^]. [19] As shown, subjects with LA dilation were older, less likely to be male, and more likely to be hypertensive (p<0.01), but were otherwise clinically similar to those without LA dilation. Body size indices, whether measured by height or weight alone or by aggregate body surface area, were smaller among subjects with LA dilation (all p<0.001). Regarding imaging parameters, LA size on CMR was, on average, 55% larger among subjects with (17±2 cm^2^/m^2^), as compared to those without (11±2 cm^2^/m^2^) LA dilation. [19] LA dilation was associated with global changes in LV structure and function, as evidenced by greater impairment of LVEF and more advanced chamber dilation (all p<0.001).

**Table 1:** Clinical and Imaging Characteristics in Relation to the Presence or Absence of Left Atrial Dilation.

Parameter	Overall(n = 342)	Left Atrial Dilation +(n = 66)	Left Atrial Dilation −(n = 276)	P
**CLINICAL**				
** Age (years)**	60±12	67±11	58±12	**<0.001**
** Male gender**	79% (271)	65% (43)	83% (228)	**0.002**
** Anthropomorphic Indices**				
Height (cm)	172±10	167±10	173±10	**<0.001**
Weight (kg)	83±20	72±15	85±20	**<0.001**
BSA (cm2)	2.0±0.2	1.8±0.2	2.0±0.2	**<0.001**
** Coronary Artery Disease Risk Factors**				
Hypertension	58% (199)	77% (51)	54% (148)	**<0.001**
Hypercholesterolemia	54% (186)	56% (37)	54% (149)	0.76
Diabetes Mellitus	33% (114)	36% (24)	33% (90)	0.56
Tobacco Use	44% (152)	55% (36)	42% (116)	0.07
Family History	18% (63)	9% (6)	21% (57)	**0.03**
**IMAGING**				
** Cardiac Magnetic Resonance**				
*** Left Ventricle***				
Ejection fraction (%)	41±16	30±12	43±16	**<0.001**
Infarct Size (% LV myocardium)	17±12	17±12	16±11	0.98
End-diastolic volume (ml)	188±68	231±77	178±62	**<0.001**
ml/m^2^	97±34	127±37	89±29	**<0.001**
End-systolic volume (ml)	118±70	166±74	107±63	**<0.001**
ml/m^2^	61±36	91±37	54±31	**<0.001**
Stroke volume (ml)	70±20	66±18	71±20	**0.048**
Myocardial mass (gm)	162±56	184±54	157±55	**<0.001**
gm/m^2^	83±27	101±27	79±25	**<0.001**
*** *** ***Left Atrium***				
Diameter (cm)	3.9±0.6	4.5±0.6	3.8±0.6	**<0.001**
Area (cm^2^)	24±6	31±5	22±4	**<0.001**
cm^2^/m^2^	12±3	17±2	11±2	**<0.001**

Left atrial dilation defined using established CMR normative cutoff (>15 cm^2^/m^2^) [19].

Numbers in boldface indicate p values <0.05.

### Electrocardiographic Markers of Left Atrial Dilation

**Table 2** presents multiple indices of ECG-quantified P wave morphology in lead V1 stratified in relation to LA dilation on CMR. Mean P wave amplitude, duration, net area and terminal negative component area were all significantly greater in patients with LA dilation. Total P wave area, as quantified in lead V1, was 59% greater among patients with, as compared to those without, CMR-evidenced LA dilation, paralleling magnitude of difference in actual CMR-evidenced chamber size between groups. P wave area increased in association with prevalence of abnormal P wave morphology as classified using an established binary criterion (algebraic product of negative terminal amplitude and duration ≥40 mm•msec in lead V1). [20]–[22] However, whereas abnormal P wave morphology was associated with LA dilation in univariate analysis (OR 2.05 [CI 1.14–3.71], p = 0.02), forced entry of both P wave area and P wave morphology into a multivariate model demonstrated only P wave area to be positively associated with LA dilation.

**Table 2:** Lead V1 P wave Measurements in Relation to the Presence or Absence of Left Atrial Dilation.

A. Amplitude				
Variable	Left Atrial Dilation +	Left Atrial Dilation −	Δmean(95% CI)	P
Amplitude (µV)	47.3±42.6	34.4±27.4	12.9 (2.0, 23.9)	**0.02**
Duration (ms)	114.8±16.0	107.7±17.1	7.2 (2.6, 11.8)	**0.002**
Area (mVm·sec)	4.3±2.5	2.7±1.3	1.5 (0.9, 2.2)	**<0.001**
Negative Component Area (mV·msec)	3.2±2.6	2.0±1.3	1.2 (0.5, 1.8)	**0.001**

Left atrial dilation defined using established CMR normative cutoff (>15 cm^2^/m^2^) [19].

**Figure 2:**
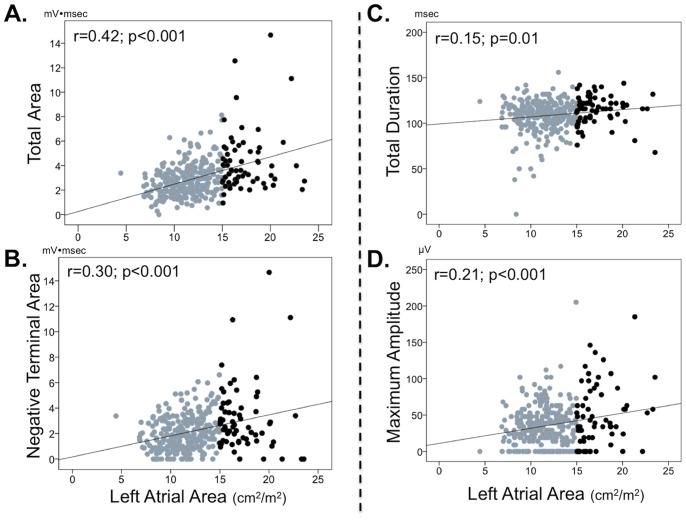
Left Atrial Size. Scatter plots relating CMR-quantified LA size to P wave morphology as measured in ECG lead V1. Data presented for total P wave area (**1A**), negative terminal area (**1B**), duration (**1C**), and maximum amplitude (**1D**). Patient data stratified based on normal (grey) and dilated (black) left atrial size on cine-CMR using an established diagnostic cutoff (>15 cm^2^/m^2^) [19].

**Figure 2** provides scatterplots for P wave indices in relation to LA size. As shown, P wave area yielded the highest correlation with LA size (r = 0.42, p<0.001), although heteroscedasticity was present for this correlation (p<0.001), as well as for other ECG variables. Moreover, despite larger body size indices (which might be expected to attenuate measures of P wave dimensions)[23]–[24] among patients without LA dilation, P wave area yielded similar magnitude of correlation with both unadjusted (r = 0.41, p<0.001) and body surface area indexed (r = 0.42, p<0.001) left atrial area, with similarly minimal impact of body size adjustment for all other P wave indices. P wave area in lead V1 remained significantly associated with LA area (p<0.001) on CMR in both forced and stepwise linear regression analyses that controlled for P wave area in all other surface ECG leads.

### Physiologic Indices in Relation to P Wave Area

#### Mitral regurgitation

P wave area in V1 was tested in relation to MR severity on cine-CMR in all patients. Echo, performed within 7 days of ECG in 86% of the study population (n = 293), was used as an independent test for MR.

**Figure 3:**
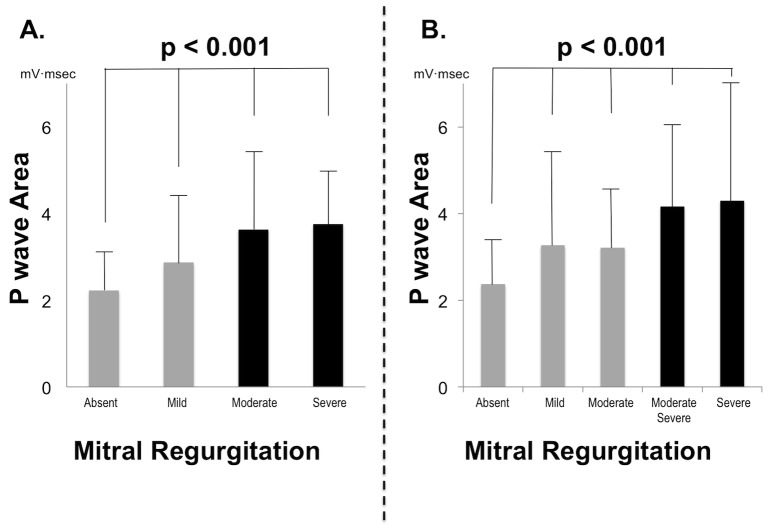
Mitral Regurgitation Severity. P wave area in lead V1 (mean ± standard deviation) in relation to MR severity on cine-CMR (**3A**) and echo (**3B**). Note that P wave area increased stepwise in relation to MR severity as measured by both modalities, with greatest magnitude of increase at a threshold of moderate-severe MR (black bars).

**Figure 3A** stratifies P wave area in relation to graded MR severity. Results demonstrate stepwise P wave area increases in relation to increasing MR on cine-CMR (p<0.001 for trend). Tukey’s post-hoc testing revealed that all cine-CMR MR categories differed significantly with respect to P wave area (p<0.05), with exception of comparison between patients with moderate vs. those with severe MR (p = 0.98). Among the aggregate population, P wave area was nearly 1.5-fold higher among patients with MR graded as moderate or severe (3.7±1.9), as compared to those with mild or absent MR (2.6±1.5, p<0.001).

**Figure 3B** stratifies P wave area in relation to MR severity on echo. Echo similarly demonstrated P wave area to increase in relation to graded severity of MR (p<0.001 for trend). Tukey’s post-hoc testing demonstrated significant differences in P wave area between patients with no MR as compared to each echo MR grade (all p≤0.01), although comparisons between MR affected patients based on echo-assigned grade did not achieve significance (p = NS).

#### Pulmonary arterial pressure

**Figure 4:**
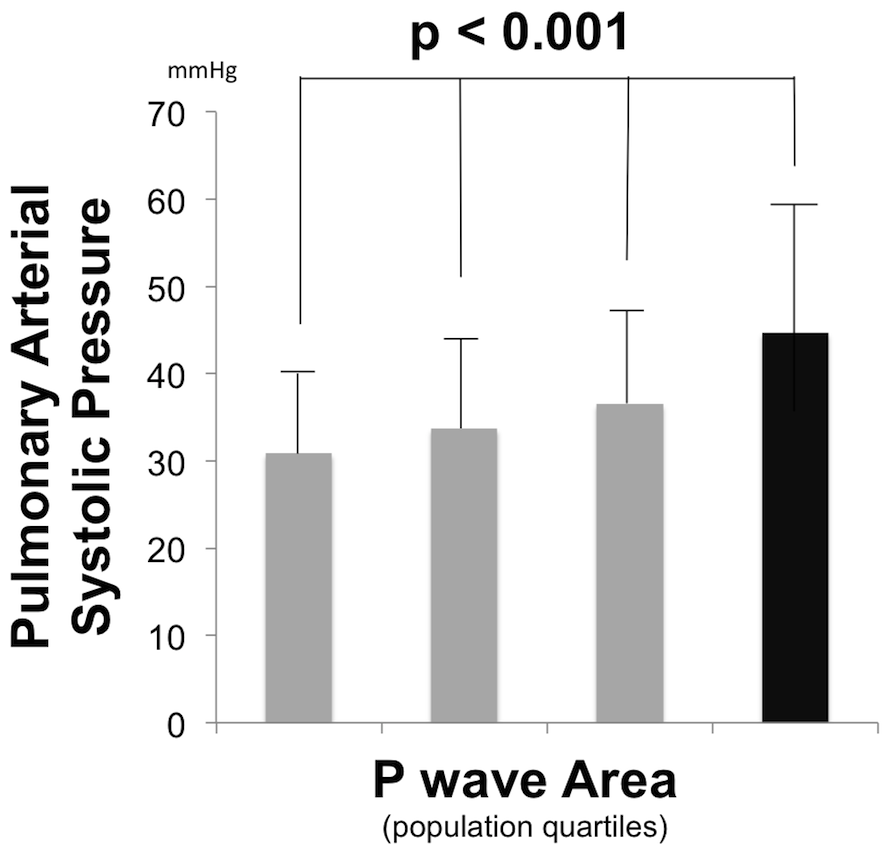
Pulmonary Arterial Pressure. Pulmonary arterial systolic pressure (mean ± standard deviation) among population subgroups stratified based on lead V1 P wave area quartiles (≤1.92 | 1.93–2.70 | 2.71–3.65 | >3.65 mV·msec). Black bar = top P wave area quartile.

**Figure 4** examines mean PA systolic pressure, as measured by echo, among population-based quartiles stratified based on P wave area. As shown, PA pressure increased in association with P wave area (p<0.001 for trend), including a near 50% increase in mean PA systolic pressure among the highest (45±14 mmHg) as compared to the lowest (31±9 mmHg) P wave area quartile. Consistent with this, Tukey’s post-hoc testing demonstrated significant differences between patients in the top P wave area quartile vs. all other groups (all p<0.01).

### Markers of Increased P Wave Area

Logistic regression was used to assess the contribution of clinical and imaging variables to increased P wave area. Regarding clinical variables, patients in the highest quartile of P wave area (≥3.5 mV·msec) were more likely to be hypertensive (29% vs. 17%, p = 0.008) as compared to the remainder of the population, but were otherwise clinically similar with regard to age, gender, and prevalence of diabetes mellitus (all p = NS). Accordingly, hypertensive status, together with LA size as well as physiologic indices (MR, PA pressure) associated with P wave area in univariate analysis were tested in CMR and echo-specific multivariate logistic regression models.

**Tables 3** **–** **4** present multivariate models incorporating imaging variables from CMR (**3**) and echo (**4**). As shown, being in the upper quartile of P wave area was independently associated with LA size measured by either modality even after controlling for MR severity and hypertensive status. Applied clinically, results demonstrate that each 5 cm^2^/m^2^ increase in LA area by CMR resulted in a two-fold increased in likelihood of increased (i.e. top quartile) P wave area by ECG (odds ratio [OR] 1.21 per cm^2^/m^2^, p<0.001), with findings similar for echo-quantified left atrial diameter (OR 3.03 per cm/m^2^, p = 0.03). Regarding functional indices, echo results demonstrated PA systolic pressure to be independently associated with increased P wave area (OR 1.49 per 10 mmHg, p = 0.01) even after controlling for LA diameter and graded severity of MR.

**Table 3:** Multivariate Models for Prediction of Increased P wave Area in Lead V1* in Overall CMR Population.

*Model chi-square = 48.08, p<0.001*			
Variable	Odds Ratio	95% Confidence Interval	P
**Left Atrial Area** (per cm^2^/m^2^)	1.21	1.10–1.34	**<0.001**
**Mitral Regurgitation** (CMR graded severity [0–III])	1.46	1.06–2.01	**0.02**
**Hypertension**	1.39	0.78–2.48	0.27

**Table 4:** Multivariate Models for Prediction of Increased P wave Area in Lead V1* in Echocardiography Sub-group.

*Model chi-square = 33.85, p<0.001*			
Variable	Odds Ratio	95% Confidence Interval	P
**Left Arial Diameter** (per cm/m^2^)	3.03	1.13–8.12	**0.03**
**Pulmonary Arterial Systolic Pressure** (per 10 mmHg)	1.49	1.14–1.86	**0.01**
**Mitral Regurgitation** (echo graded severity [0–IV])	1.38	0.84–2.27	0.20
**Hypertension**	1.04	0.45–2.42	0.93

(*top P wave area quartile ≥3.5 mV·msec).

### Clinical Endpoints

Clinical follow-up was available in 64% (n = 218) of the study population. Study participants with follow-up were similar to those without follow-up with respect to age, gender, P wave area on ECG, and LA size on cine-CMR (all p = NS).

**Figure 5:**
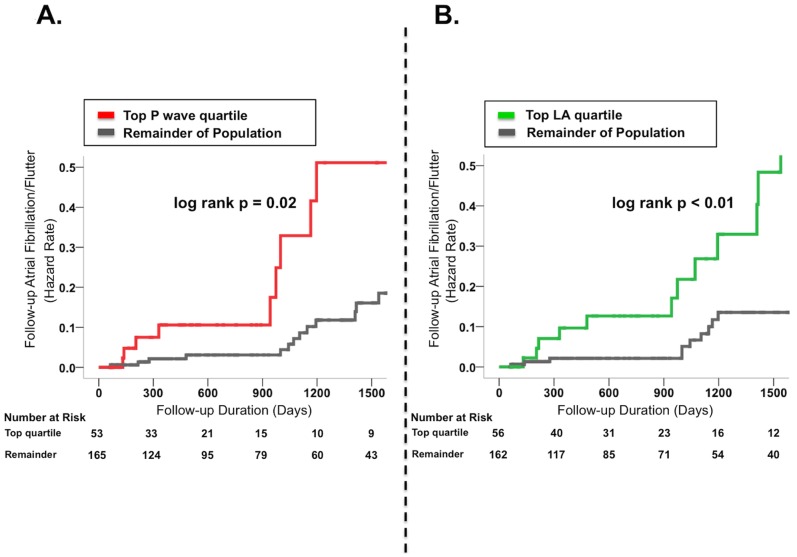
Atrial Fibrillation/Flutter Risk as Stratified by LA Remodeling Indices. Kaplan-Meier plots relating baseline P wave area (5A) and body surface area indexed LA area (5B) to follow-up risk for AF/AFl. Note that both ECG and CMR indices demonstrated increased risk for AF/AFl among patients in the highest quartile of LA remodeling.

During a mean interval of 2.4±1.9 years following ECG, 11.5% (n = 25) of patients experienced atrial fibrillation (AF) or flutter (AFl). Incidence of the composite endpoint of AF/AFl increased in relation to P wave area as well as LA size, and was 4-fold higher among patients in the highest, as compared to the lowest, P wave area quartile (17.0% vs. 3.9%, p = 0.03), with similar magnitude of difference when patient groups were compared based on quartiles of LA area (23.2% vs. 6.9%, p = 0.01). As shown in **Figure 5**, both indices stratified arrhythmic risk (p<0.05). In Cox regression analysis, patients within the top quartile of P wave area manifested a 2.6-fold increase in risk for incident atrial fibrillation/flutter as compared to the remainder of the population (95% CI 1.1 to 5.9, p = 0.02), whereas patients in the top quartile of LA area index manifested a 3.2-fold (95% CI 1.4 to 7.0, p = 0.005) increase in risk. Similarly, when examined as continuous variables in univariate Cox regression models, both a one standard deviation increase in P wave area (1.54 mV·msec) as well as body surface area indexed LA area (3.19 cm^2^/m2) conferred similar risk for incident AF/AFl during longitudinal follow-up: [P wave area hazard ratio 1.57 (95% CI 1.2–2.1), p = 0.003/LA area hazard ratio 1.64 (CI 1.2–2.3), p = 0.005].

## Discussion

This study, the first to test quantitative ECG assessment of LA remodeling in relation to both physiologic indices and clinical arrhythmic risk, offers several key findings: First, P wave area – as measured on routine surface ECG in lead V1– parallels LA size on CMR. Second, LA loading conditions influence P wave area, as evidenced by stepwise increases in relation to increasing graded MR severity. Association between P wave area and MR severity on CMR was independent of LA chamber size, supporting the concept that P wave area provides a composite marker of atrial stretch, rather than a simple index of chamber size alone. Finally, P wave area stratified longitudinal risk for atrial arrhythmias: risk of incident AF/AFl was nearly 3-fold higher among patients in the top quartile of P wave area as compared to the remainder of the population (hazard ratio [HR] 2.6, CI 95% 1.1 to 5.9, p = 0.02), paralleling a similar magnitude of risk among patients in the top quartile of LA area (HR 3.2, CI 1.4 to 7.0, p = 0.005).

Our findings build upon prior research concerning the link between ECG-evidenced P wave morphology and LA size. Established ECG criteria (such as P wave duration >0.11 sec, V1 negative terminal component >40 msec·mm, and prominent notching) have individually been shown to yield wide ranges of sensitivity (12 to 96%) and specificity (64 to 100%) for echo-evidenced LA dilation, possibly reflecting differences in study population, individual ECG criteria, and echo cutoffs for chamber enlargement. [9]–[11] CMR, which provides excellent endocardial definition and more reproducible chamber quantification than echo, [25] has also been used as a comparator for ECG. Consistent with prior echo studies, Tsao et al. reported that different ECG criteria yielded marked variability for assessment of CMR-evidenced LA enlargement (sensitivity 8 to 94%, specificity 21 to 90%). [26] However, despite differences in imaging modality, such prior studies share a common approach in that P wave data has been generally used to categorize LA dilation in a binary fashion, rather than to assess LA size as a continuous parameter. As evidenced in our study (**Figure 1**), P wave morphology can vary among patients with LA dilation, thereby compromising utility of established morphological criteria for LA enlargement, such as those predicated on P wave axis or terminal deflection component. Our results demonstrate that P wave area, a composite index of amplitude and duration, provides a continuous index of LA remodeling, reflecting physiologic changes in LA geometry.

It is important to recognize that whereas P wave area yielded higher correlation (r = 0.45) with LA size than did other P wave indices (r = 0.15–0.3), heteroscedasticity was present for all ECG indices. Limited correlation between P wave indices and LA area is consistent with our finding (**Tables 3** **–** **4**) that P wave area reflects a composite manifestation of both LA size and pressure volume/loading conditions, rather than a singular index of LA size alone. We note that our observed associations between P wave area and MR severity on CMR as well as estimated PA pressures on echo are consistent with prior physiologic studies that have examined the impact of both loading conditions and tissue properties on LA conductivity. Among patients with heart failure, LA dilation has been found to be accompanied by low voltage amplitudes on invasive electro-anatomic mapping, and prolonged P wave duration on surface ECG. [27] Similar findings have also been reported in conditions associated with LA remodeling, including mitral stenosis and atrial septal defect. [28]–[29] Changes in atrial conductivity have been shown to reflect alterations in tissue substrate. In a canine model of pacing-induced heart failure, regions of slow atrial conductivity had histology-evidenced interstitial fibrosis. [30] Atrial tissue characterization using delayed enhancement CMR has been used to demonstrate that areas of low voltage on electro-anatomic mapping correspond to regions of atrial fibrosis. [31] Of note, reverse LA remodeling has been shown to be capable of improving LA electrical properties. For example, among patients with mitral stenosis, commissurotomy has been shown to reduce both LA pressures and volumes, resulting in decreased P wave duration on ECG. [32] Taken together, these data demonstrate that that LA tissue substrate, geometry, and loading conditions all bear potential influence on ECG waveform indices, supporting the notion that P wave morphology offers a composite index for LA arrhythmic substrate.

Regarding clinical implications, our findings are consistent with prior literature linking P wave morphology to arrhythmic risk. Among 1550 subjects in the Framingham Heart Study, those in the top 5 percent of P wave duration had a 2.5-fold increase in relative risk for AF compared to the remainder of the population. [33] In the ARIC study, increased P wave duration (HR 3.9) and area (HR 2.8) were each associated with AF even after controlling for age, gender, and clinical risk factors for CAD. [34] Of importance, neither study examined the predictive value of P wave indices in relation to imaging parameters of LA dilation, which is itself a well-established predictor for AF. [5]–[7] Our results demonstrate the predictive value of P wave area to be near equivalent to that of CMR-quantified LA area for prediction of AF/AFl (HR 2.6 to 3.2). Applied clinically, these findings suggest that P wave data acquired from standard ECG, which is both inexpensive and widely available, can offer equivalent risk stratification for AF to that of sophisticated imaging via CMR.

Several limitations should be noted. First, whereas both ECG and CMR were performed within a narrow interval (mean Δ 0.1±1.4 days), LA size itself can change dynamically due to transient alterations in loading conditions. Thus, differences between modalities may in part reflect interim changes in LA geometry. Second, whereas P wave area was tested in relation to a clinical endpoint of AF/AFl, event rates were captured based on clinical documentation, raising the possibility that clinically silent episodes were not captured via longitudinal follow-up. On the other hand, this potential source of ascertainment bias would not be expected to vary in relation to LA geometry as assessed by either ECG or imaging, and would therefore not impact our observed association between P wave area, LA geometry, and arrhythmic risk. Third, the number of clinical endpoints in this study were relatively low (n = 25), prohibiting definite conclusions regarding stratification of longitudinal risk for AF/AFl. Larger scale, population-based studies inclusive of subjects without ischemic heart disease or LV systolic dysfunction are necessary for more definitive assessment of P wave area for assessment of LA remodeling and stratification of atrial arrhythmic risk.

In conclusion, the current study demonstrates that ECG-quantified P wave area provides an index of LA remodeling that parallels CMR-evidenced LA chamber geometry and provides similar predictive value to imaging for stratification of longitudinal risk for AF/AFl among subjects with ischemic heart disease. Future studies are warranted to test this in other cohorts at risk for LA remodeling, such as subjects with non-ischemic cardiomyopathy, and to ascertain whether serial changes in P wave area may provide additional insights into changing LA chamber geometry, pressure and arrhythmic risk over time.

## References

[1] Tsang TS, Barnes ME, Gersh BJ, Bailey KR, Seward JB (2002) Left atrial volume as a morphophysiologic expression of left ventricular diastolic dysfunction and relation to cardiovascular risk burden. Am J Cardiol 90: 1284–1289. 12480035 10.1016/s0002-9149(02)02864-3

[2] Gerdts E, Oikarinen L, Palmieri V, Otterstad JE, Wachtell K, et al (2002) Correlates of left atrial size in hypertensive patients with left ventricular hypertrophy: the Losartan Intervention For Endpoint Reduction in Hypertension (LIFE) Study. Hypertension 39: 739–743. 11897755 10.1161/hy0302.105683

[3] Gardin JM, McClelland R, Kitzman D, Lima JA, Bommer W, et al (2001) M-mode echocardiographic predictors of six- to seven-year incidence of coronary heart disease, stroke, congestive heart failure, and mortality in an elderly cohort (the Cardiovascular Health Study). Am J Cardiol 87: 1051–1057. 11348601 10.1016/s0002-9149(01)01460-6

[4] Benjamin EJ, D’Agostino RB, Belanger AJ, Wolf PA, Levy D (1995) Left atrial size and the risk of stroke and death. The Framingham Heart Study. Circulation 92: 835–841. 7641364 10.1161/01.cir.92.4.835

[5] Vaziri SM, Larson MG, Benjamin EJ, Levy D (1994) Echocardiographic predictors of nonrheumatic atrial fibrillation. The Framingham Heart Study. Circulation 89: 724–730. 8313561 10.1161/01.cir.89.2.724

[6] Berruezo A, Tamborero D, Mont L, Benito B, Tolosana JM, et al (2007) Pre-procedural predictors of atrial fibrillation recurrence after circumferential pulmonary vein ablation. Eur Heart J 28: 836–841. 17395676 10.1093/eurheartj/ehm027

[7] Olshansky B, Heller EN, Mitchell LB, Chandler M, Slater W, et al (2005) Are transthoracic echocardiographic parameters associated with atrial fibrillation recurrence or stroke? Results from the Atrial Fibrillation Follow-Up Investigation of Rhythm Management (AFFIRM) study. J Am Coll Cardiol 45: 2026–2033. 15963405 10.1016/j.jacc.2005.03.020

[8] Lewis T (1911) The Electrocardiographic Method and its Relationship to Clinical Medicine. Proc R Soc Med 4: 81–100. 10.1177/003591571100400606 PMC2004300 19975235

[9] Munuswamy K, Alpert MA, Martin RH, Whiting RB, Mechlin NJ (1984) Sensitivity and specificity of commonly used electrocardiographic criteria for left atrial enlargement determined by M-mode echocardiography. Am J Cardiol 53: 829–832. 6230922 10.1016/0002-9149(84)90413-2

[10] Miller DH, Eisenberg RR, Kligfield PD, Devereux RB, Casale PN, et al (1983) Electrocardiographic recognition of left atrial enlargement. J Electrocardiol 16: 15–22. 6220099 10.1016/s0022-0736(83)80154-x

[11] Waggoner AD, Adyanthaya AV, Quinones MA, Alexander JK (1976) Left atrial enlargement. Echocardiographic assessment of electrocardiographic criteria. Circulation 54: 553–557. 134852 10.1161/01.cir.54.4.553

[12] Kochav JD, Okin PM, Wilson S, Afroz A, Renilla A, et al (2013) Usefulness of Q-wave area for threshold-based stratification of global left ventricular myocardial infarct size. Am J Cardiol 112: 174–180. 23611753 10.1016/j.amjcard.2013.03.013 PMC3878979

[13] Okin PM, Roman MJ, Devereux RB, Pickering TG, Borer JS, et al (1998) Time-voltage QRS area of the 12-lead electrocardiogram: detection of left ventricular hypertrophy. Hypertension 31: 937–942. 9535418 10.1161/01.hyp.31.4.937

[14] Lang RM, Biereg M, Devereux RM, Flachskampf FA, Foster E, et al (2005) Recommendations for Chamber Quantification: A Report from the American Society of Echocardiography’s Guidelines and Standards Committee and the Chamber Quantification Writing Group, Developed in Conjunction with the European Association of Echocardiography, a Branch of the European Society of Cardiology. J Am Soc Echocardiogr 18: 1440–1463. 16376782 10.1016/j.echo.2005.10.005

[15] Heitner J, Bhumireddy GP, Crowley AL, Weinsaft J, Haq SA, et al (2012) Clinical application of cine-MRI in the visual assessment of mitral regurgitation compared to echocardiography and cardiac catheterization. PLoS One 7: e40491. 22815751 10.1371/journal.pone.0040491 PMC3398949

[16] Lauren A, Simprini AA, Klem I, Jensen CJ, Kim RJ, et al.. (2013) Routine cine-CMR for assessment of prosthesis-associated mitral regurgitation - a multicenter, multivendor study. Journal of Cardiovascular Magnetic Resonance 2013.

[17] Jones EC, Devereux RB, Roman MJ, Liu JE, Fishman D, et al (2001) Prevalence and correlates of mitral regurgitation in a population-based sample (the Strong Heart Study). Am J Cardiol 87: 298–304. 11165964 10.1016/s0002-9149(00)01362-x

[18] Zoghbi WA, Enriquez-Sarano M, Foster E, Grayburn PA, Kraft CD, et al (2003) Recommendations for evaluation of the severity of native valvular regurgitation with two-dimensional and Doppler echocardiography. J Am Soc Echocardiogr 16: 777–802. 12835667 10.1016/S0894-7317(03)00335-3

[19] Maceira AM, Cosin-Sales J, Roughton M, Prasad SK, Pennell DJ (2010) Reference left atrial dimensions and volumes by steady state free precession cardiovascular magnetic resonance. J Cardiovasc Magn Reson 12: 65. 21070636 10.1186/1532-429X-12-65 PMC2994941

[20] Morris JJ Jr, Estes EH Jr, Whalen RE, Thompson HK Jr, McIntosh HD (1964) P-Wave Analysis in Valvular Heart Disease. Circulation 29: 242–252. 14119389 10.1161/01.cir.29.2.242

[21] Chirife R, Feitosa GS, Frankl WS (1975) Electrocardiographic detection of left atrial enlargement. Correlation of P wave with left atrial dimension by echocardiography. Br Heart J 37: 1281–1285. 131563 10.1136/hrt.37.12.1281 PMC482954

[22] Termini BA, Lee YC (1975) Echocardiographic and electrocardiographic criteria for diagnosing left atrial enlargement. South Med J 68: 161–165. 123358 10.1097/00007611-197502000-00010

[23] Nasir JM, Rubal BJ, Jones SO, Shah AD (2012) The effects of body mass index on surface electrocardiograms in young adults. J Electrocardiol 45: 646–651. 23021815 10.1016/j.jelectrocard.2012.07.022

[24] Maunganidze F, Woodiwiss AJ, Libhaber CD, Maseko MJ, Majane OH, et al (2013) Obesity markedly attenuates the validity and performance of all electrocardiographic criteria for left ventricular hypertrophy detection in a group of black African ancestry. J Hypertens 31: 377–383. 23169235 10.1097/HJH.0b013e32835b8daa

[25] Grothues F, Smith GC, Moon JC, Bellenger NG, Collins P, et al (2002) Comparison of interstudy reproducibility of cardiovascular magnetic resonance with two-dimensional echocardiography in normal subjects and in patients with heart failure or left ventricular hypertrophy. Am J Cardiol 90: 29–34. 12088775 10.1016/s0002-9149(02)02381-0

[26] Tsao CW, Josephson ME, Hauser TH, O’Halloran TD, Agarwal A, et al (2008) Accuracy of electrocardiographic criteria for atrial enlargement: validation with cardiovascular magnetic resonance. J Cardiovasc Magn Reson 10: 7. 18272008 10.1186/1532-429X-10-7 PMC2244611

[27] Sanders P, Morton JB, Davidson NC, Spence SJ, Vohra JK, et al (2003) Electrical remodeling of the atria in congestive heart failure: electrophysiological and electroanatomic mapping in humans. Circulation 108: 1461–1468. 12952837 10.1161/01.CIR.0000090688.49283.67

[28] John B, Stiles MK, Kuklik P, Chandy ST, Young GD, et al (2008) Electrical remodelling of the left and right atria due to rheumatic mitral stenosis. Eur Heart J 29: 2234–2243. 18621772 10.1093/eurheartj/ehn329

[29] Roberts-Thomson KC, John B, Worthley SG, Brooks AG, Stiles MK, et al (2009) Left atrial remodeling in patients with atrial septal defects. Heart Rhythm 6: 1000–1006. 19493703 10.1016/j.hrthm.2009.03.050

[30] Li D, Fareh S, Leung TK, Nattel S (1999) Promotion of atrial fibrillation by heart failure in dogs: atrial remodeling of a different sort. Circulation 100: 87–95. 10393686 10.1161/01.cir.100.1.87

[31] Oakes RS, Badger TJ, Kholmovski EG, Akoum N, Burgon NS, et al (2009) Detection and quantification of left atrial structural remodeling with delayed-enhancement magnetic resonance imaging in patients with atrial fibrillation. Circulation 119: 1758–1767. 19307477 10.1161/CIRCULATIONAHA.108.811877 PMC2725019

[32] John B, Stiles MK, Kuklik P, Brooks AG, Chandy ST, et al (2010) Reverse remodeling of the atria after treatment of chronic stretch in humans: implications for the atrial fibrillation substrate. J Am Coll Cardiol 55: 1217–1226. 20298929 10.1016/j.jacc.2009.10.046

[33] Magnani JW, Johnson VM, Sullivan LM, Gorodeski EZ, Schnabel RB, et al.. (2011) P wave duration and risk of longitudinal atrial fibrillation in persons >/ = 60 years old (from the Framingham Heart Study). Am J Cardiol 107: 917–921 e911. 10.1016/j.amjcard.2010.10.075 PMC3049849 21255761

[34] Soliman EZ, Prineas RJ, Case LD, Zhang ZM, Goff DC Jr (2009) Ethnic distribution of ECG predictors of atrial fibrillation and its impact on understanding the ethnic distribution of ischemic stroke in the Atherosclerosis Risk in Communities (ARIC) study. Stroke 40: 1204–1211. 19213946 10.1161/STROKEAHA.108.534735 PMC2685189

